# Usnic Acid Isolated from *Usnea antarctica* (Du Rietz) Reduced In Vitro Angiogenesis in VEGF- and bFGF-Stimulated HUVECs and Ex Ovo in Quail Chorioallantoic Membrane (CAM) Assay

**DOI:** 10.3390/life12091444

**Published:** 2022-09-16

**Authors:** Klaudia Petrová, Miriam Bačkorová, Zuzana Demčišáková, Eva Petrovová, Michal Goga, Mária Vilková, Richard Frenák, Martin Bačkor, Ján Mojžiš, Martin Kello

**Affiliations:** 1 Department of Pharmacology, Faculty of Medicine, Pavol Jozef Šafárik University, 040 01 Košice, Slovakia; 2 Department of Pharmaceutical Technology, Pharmacognosy and Botany, University of Veterinary Medicine and Pharmacy, 041 81 Košice, Slovakia; 3 Department of Morphological Disciplines, University of Veterinary Medicine and Pharmacy, 041 81 Košice, Slovakia; 4 Department of Botany, Institute of Biology and Ecology, Faculty of Science, Pavol Jozef Šafárik University, Mánesova 23, 041 67 Košice, Slovakia; 5 NMR Laboratory, Department of Chemistry, Faculty of Science, Pavol Jozef Šafárik University, Moyzesova 11, 040 01 Košice, Slovakia; 6 Institute of Biotechnology, Faculty of Biotechnology and Food Sciences, Slovak University of Agriculture in Nitra, Trieda Andreja Hlinku 2, 949 76 Nitra, Slovakia

**Keywords:** angiogenesis, bFGF, CAM, HUVECs, usnic acid, VEGF

## Abstract

**Simple Summary:**

Anti-angiogenic therapy, a promising strategy against cancer progression, is limited by drug resistance. Natural plants, such as secondary metabolites of lichens, may represent an appropriate strategy to increase the effectiveness of conventional therapies and overcome resistance to anti-angiogenic therapy if combined with existing chemotherapy. Accordingly, our study was designed to determine the potential anti-angiogenic effect of usnic acid, a secondary metabolite of lichens, on VEGF- and bFGF-stimulated HUVECs as well as in quail chorioallantoic membrane assays, which were supplemented by histological sections of CAM-affected layers.

**Abstract:**

Natural products include a diverse set of compounds of drug discovery that are currently being actively used to target tumor angiogenesis. In the present study, we evaluated the anti-angiogenic activities of secondary metabolite usnic acid isolated from *Usena antarctica.* We investigated the in vitro effects on proliferation, migration, and tube formation of VEGF- and bFGF-stimulated HUVECs. Ex ovo anti-angiogenic activity was evaluated using the CAM assay. Our findings demonstrated that usnic acid in the concentration of 33.57 µM inhibited VEGF (25 ng/mL) and bFGF (30 ng/mL)-induced HUVECs proliferation, migration, and tube formation. The ex ovo CAM model was used to confirm the results obtained from in vitro studies. VEGF- and bFGF-induced vessel formation was inhibited by usnic acid after 72 h in over 2-fold higher concentrations compared to in vitro. Subsequently, histological sections of affected chorioallantoic membranes were stained with hematoxylin–eosin and alcian blue to determine the number and diameter of vessels as well as the thickness of the individual CAM layers (ectoderm, mesoderm, endoderm). Usnic acid was able to suppress the formation of VEGF- and bFGF-induced vessels with a diameter of less than 100 μm, which was demonstrated by the reduction of mesoderm thickness as well.

## 1. Introduction

Angiogenesis, the formation and remodeling of new capillaries from pre-existing vasculature [1], has always been the topic of major scientific interest in the field of tumor growth and metastasis [2]. It is a complex multistep process, which involves extensive interactions between endothelial cells, soluble factors, and components of the extracellular matrix (ECM) [3]. Dr. Judah Folkman first initiated the concept of tumor angiogenesis in 1971. He presented a hypothesis that malignant tumor growth depends on angiogenesis, and that suppression of angiogenesis may be therapeutically significant. The hypothesis predicted that the tumor cannot exceed the microscopic size of 1–2 mm^3^ in an avascular state [4,5]. Moreover, Dr. Folkman also isolated factors from tumors that induce angiogenesis [6] and then considered that inhibition of those pro-angiogenic markers can block new vessel formation [7]. Much attention has been focused on one of the most specific and critical regulators of angiogenesis, the vascular endothelial growth factor (VEGF) [8]. Several other growth factors are implicated in the formation of a defective vascular network in tumors, such as members of the angiopoietins, platelet-derived growth factor (PDGF), transforming growth factor (TGF-B) families, and basic fibroblast growth factor (bFGF) [9]. All these factors play pivotal roles in the development of tumor angiogenesis by stimulating endothelial cell proliferation, migration, and capillary tube formation [10]. Based on this information, it is clear that inhibition of angiogenesis is an important strategy for the treatment of solid tumors [11].

The use of current anti-angiogenic therapeutics faces several barriers, including resistance to chemotherapy. Chemotherapy in combination with natural products may represent an appropriate way to improve the efficacy of conventional therapeutics and may be used to overcome resistance to anti-angiogenic therapy [12,13]. Natural products are considered a rich reservoir of bioactive compounds with therapeutic potential. It is estimated that between 1981 and 2019, approximately 25% of all newly approved anticancer drugs were nature-derived compounds [14,15].

Secondary metabolites of lichens have gained importance as anti-cancer agents targeting angiogenesis. Few studies have been focused on the potential anti-angiogenic effects of lichen’s secondary metabolites. Several lichen-derived molecules have been shown to have significant anti-angiogenic activity with low toxicity. These metabolites, such as emodin, vulpinic acid, barbatolic acid, parietin, olivetoric acid, and usnic acid, have been shown to inhibit several important steps of the angiogenic process [16,17,18,19,20,21].

Previous studies reported that Antarctic lichens show stronger antioxidant activity compared to tropical or temperate zone lichens [22,23]. Authors suggested that extreme Antarctic conditions, such as low temperature, winter darkness, high UV-B, and solar irradiation may increase oxidative stress. Therefore, Antarctic lichens have larger amounts of antioxidant substances and have higher antioxidant activity.

Usnic acid (UA), as one of the few commercially available metabolites of lichens, is the main focus of our research. To date, antibacterial, antiviral, antioxidant, antifungal, and anticancer effects of tropical or temperate zone UA have been proved [24,25,26,27,28]. Furthermore, UA has been formulated as a pure substance in creams, toothpaste, mouthwashes, deodorants, and sun protection products [26]. In the process of angiogenesis, UA interferes with multiple signal transduction pathways and thus limits proliferation and metastasis [17]. To the best of our knowledge, there is no information about the anti-angiogenic activity of Antarctic UA.

UA also plays an important role as an antioxidant, as mentioned above. The most commonly tested compounds as antioxidants are butylated hydroxyanisole (BHA), butylated hydroxytoluene (BHT), tertbutylhydroquinone (TBHQ), and propyl gallate (PG). All of these antioxidants are made by the synthetic method [29]. It is known that low cost and high efficiency are linked to synthetic antioxidants. Considering their carcinogenic potential, natural replacements are still required [30].

Accordingly, our study aimed to evaluate the potential anti-angiogenic effects of UA isolated from *Usnea antarctica* in vitro as well as ex ovo in the CAM assay, focusing on histological sections of CAM under VEGF- and bFGF-stimulated conditions.

## 2. Materials and Methods

### 2.1. Chemicals

Medium 199, penicillin, streptomycin, and newborn calf serum (NBCS) were obtained from Lonza (Verviers, Belgium). Human serum was obtained from PAA (Pasching, Austria) and l-glutamine from Sigma-Aldrich (St. Louis, MO, USA). Vascular endothelial growth factor (VEGF) was purchased from Biosource (Camarillo, CA, USA) and basic fibroblast growth factor (bFGF) from Sigma-Aldrich. Matrigel basement membrane matrix was obtained from Becton Dickinson Labware (Bedford, MA, USA). Other materials used in the methods described below are specified in detail in related references or in the text.

### 2.2. Collection, Extraction, and Characterization of Usnic Acid

Dr. Bačkor collected and determined the lichen *Usnea antarctica* (Du Rietz) from Antarctica during an expedition in January 2017. The material was collected at James Ross Island with other cryptogams [31]. The lichen specimen was deposited in the herbarium of P. J. Šafárik in Košice.

Lichen extract of *U. antarctica* contains depsides, depsidones, lipids, and dibenzofurans [32]. Ten grams of DW (dry weight) lichen thalli were rinsed with distilled water to get rid of particles from the surface and air-dried at room temperature (26 °C). Extraction of secondary metabolites from the lichen thalli was conducted according to Solhaug and Gauslaa [33]. One milligram DW of the extract was dissolved in acetone, and a TLC plate for identification of lichen substances was performed. The ratio of mobile phase for separation of lichen compounds from *U. antarctica* by column chromatography was 2:1:0.3 (cyclohexane:ethylacetate:acetic acid). Collected fractions were used for further identification by HPLC.

Analyses of fractions were performed by semi-preparative method HPLC (Agilent Technologies 1260 Infinity II) by gradient [34] under the following conditions: A 7 μm column Kromasil SGX C18, flow rate 0.7 mL min^−1^, mobile-phase: A = H_2_O:acetonitrile:H_3_PO_4_ (80:19:1) and B = 90% acetonitrile, gradient program: 0 min 25% B, 5 min 50% B, 20 min 100% B, 25 min 25% B. Detection was performed at a wavelength of 254 nm (Agilent Technologies detector 1260 VWD). Usnic acid (UA, Aldrich Company 329967 5C) was used as a standard for confirmation of the isolated compound.

NMR spectra of isolated UA were recorded on a Varian VNMRS (599.87 MHz for ^1^H and 150.84 MHz for ^13^C) spectrometer with a 5 mm inverse-detection H-X probe equipped with a z-gradient coil at 299.15 K. All the pulse programs were taken from the Varian sequence library. Chemical shifts (*δ* in ppm) were given from the internal solvent and the partially deuterated residual—DMSO-d_6_ 39.5 ppm for ^13^C and DMSO-d_5_ 2.5 ppm for ^1^H. NMR spectra were processed and analyzed in MestReNova version 14.2.1 (Mestrelab Research, Santiago de Compostela, Spain). The purity of extracted UA was gained from quantitative NMR data.

In all experiments, UA was diluted in DMSO–dimethylsulfoxide with *v*/*v* not exceeding 0.2%.

### 2.3. Cell Culture

Primary human umbilical cord vein endothelial cells (HUVECs) were isolated from umbilical cords obtained from local hospitals under the University of Pavol Jozef Šafárik in Košice. The study was approved by the Ethical Committee of the Faculty of Pharmacy, Comenius University, in Bratislava (June 2019). After childbirth, umbilical cords were placed in cord buffer (KCl, NaCl, Hepes, d-glucose H_2_O, penicillin/streptomycin) and stored at 4 °C. Cells were freshly isolated every two weeks. The umbilical vein was washed with cord buffer, filled with collagenase type II (Grand Island, NY, USA), and incubated for 20 min at 37 °C. To isolate endothelial cells, the vein was flushed with a medium (M199, penicillin/streptomycin), and the cell suspension was centrifuged (1.000 rpm, 5 min). Afterward, HUVECs were re-suspended in growth medium cM199 (=M199 medium supplemented with 20% heat-inactivated newborn calf serum, 10% heat-inactivated human serum, 150 μg/mL crude endothelial cell growth factor (ECGF), 5 U/mL heparin, 100 IU/mL penicillin, and 100 μg/mL streptomycin) and cultured on a gelatin-coated 75 cm^2^ flask at 37 °C under 5% CO_2_/95% air atmosphere. Isolated HUVECs were then used for all experiments.

### 2.4. Methyl–Thiazole–Tetrazolium (MTT) Assay

The effect of lichen metabolite UA on the viability of HUVECs was assessed using a 3-(4,5-dimethylthiazol-2-yl)-2,5-diphenyltetrazolium bromine (MTT) reduction assay. First, HUVECs were stimulated with different VEGF and bFGF concentrations (10–40 ng/mL) to determine the optimal dose of either factor to promote endothelial cell proliferation. Subsequently, cells were plated in gelatin-coated 96-well micro-culture plates (5 × 10^3^ cells/well) containing 80 μL of medium and incubated for 24 h at 37 °C in a 5% CO_2_ atmosphere. Then 24 h after seeding, 20 μL of vehicle (0.1% DMSO) and aliquots of drug solutions (10, 50, 100 μM) in the presence or absence of VEGF (25 ng/mL) or bFGF (30 ng/mL) were added to HUVECs in triplicate wells. After 48 h of culturing, MTT reagent was added to each well at a final concentration of 0.5 mg/mL, and cells were incubated for another 4 h at 37 °C. Then 100 μL of sodium dodecyl sulfate (SDS) was added to each well, and the plate was kept on a shaker for 15 min to dissolve the formazan crystals formed in intact cells. A microplate Cytation^TM^ 3 Cell Imaging Multi-Mode Reader(Biotek, Winooski, VT, USA) was used to measure the absorbance at wavelength 595 nm. Results were expressed as the percentages of reduced MTT, assuming the absorbance of control cells as 100%.

### 2.5. Scratch Assay (Wound Healing)

The motility of HUVECs was assayed using a wound healing assay. Briefly, HUVEC cells were plated in a gelatin-coated 24-well plate at 1 × 10^5^ cells/well and incubated in the cM199 medium until a uniform monolayer was formed. A linear scratch was then made by using an SPL Scar^TM^ scratcher (SPL Life Science, Pocheon, Korea). The cells were washed three times with 1 mL of de-ionized phosphate-buffered saline (DPBS; pH 7.4) and treated with UA (33.57 μM) in the presence or absence of 25 ng/mL of VEGF and 30 ng/mL of bFGF in ECGF and heparin-free medium. At the end of the experiment, cells were fixed with methanol, stained with CellTrace^TM^ Yellow (Thermo Fisher, Rockford, IL, USA), and then captured under an inverted light microscope. The wounded area was photographed at the start (t = 0 h) and after 24 h. Images of the wounded areas were captured using a microplate Cytation^TM^ 3 Cell Imaging Multi-Mode Reader (Biotek, Winooski, VT, USA). The experiments were performed in duplicate wells and repeated three times with cells from different donors.

### 2.6. Tube Formation Assay

The day before performing the tube formation assay, a Matrigel matrix (BD Biosciences, Billerica, MA, USA) was incubated on ice overnight. On the day of the assay, 50 μL of Matrigel matrix was added to a 96-well plate and incubated at 37 °C, 5% CO_2_ for 30 min. The cells with tested compound (UA 33.57 μM) and in the presence or absence of growth factors (VEGF 25 ng/mL; bFGF 30 ng/mL) were transferred to each well containing the Matrigel matrix. The plates were incubated for 3 to 16 h according to the size of the tube. Tube formation was quantified by measuring segment numbers, total segment length, number of junctions, and number of nodes in three random x 3 magnification fields per well, using a microplate Cytation^TM^ 3 Cell Imaging Multi-Mode Reader (Biotek, Winooski, VT, USA). The data analysis was performed using ImageJ software.

### 2.7. Fibrin Bead Sprouting Assay

Cytodex^TM^ 3 (GE Healthcare, Chicago, IL, USA) microcarrier beads (0.5 g) were swollen in 50 mL DPBS and then autoclaved (121 °C, 20 min). HUVECs were coated on cytodex-3 beads at a density of 4 × 10^5^ cells/40 μL beads and incubated in suspension for 4 h with gentle mixing every 20 min. They were plated overnight on a T25 tissue culture flask. The next day, the suspension was transferred to a 15 mL conical tube and washed three times with 1 mL of M199 + penicillin/streptomycin to remove unattached cells. The cell-covered beads were re-suspended in a 2 mg/mL fibrinogen (Sigma-Aldrich St. Louis, MO, USA) solution containing 0.15 U/mL aprotinin (Sigma-Aldrich). Aliquots were mixed with thrombin (Sigma; 0.625 U/mL), distributed in 12-well plates (150 mL/well), and left to precipitate for 5–10 min. After further incubation (10 min, 37 °C, and 5% CO_2_), a medium containing 25 ng/mL VEGF or 30 ng/mL bFGF and 33.57 μM of UA was added to cover the precipitate. Bead assays were monitored for 10 days. Images of beads were captured using a microplate Cytation^TM^ 3 Cell Imaging Multi-Mode Reader (Biotek, Winooski, VT, USA) and analyzed using automatic WimSprout Image Analysis software (ibidi GmbH, Gräfelfing, Germany).

### 2.8. Ex Ovo CAM Assay

The CAM assay was performed as an ex ovo method. Fertilized quail eggs (*Coturnix japonica*; 120 specimens) were obtained from a certified farm (Mala Ida, Slovakia, 2021). After 72 h of incubation, eggs were removed from the incubator, sterilized with 70% ethanol, and transferred to a sterile box. The eggs were held horizontally, and the contents of the eggs were gently transferred to the culture dishes. Ex ovo cultures were returned to an incubator (38.2 ± 0.5 °C and 58% relative humidity) until embryonic day 6 (ED6). An ED6 autoclaved silicone ring (~6 mm thickness) was laid on the CAM surface for the deposition of the testing solution. The control group was treated with sodium chloride 0.9% (30 μL per egg), while a positive control group was treated with UA (75, 100 μM) in the presence or absence of 25 ng/mL of VEGF or 30 ng/mL of bFGF. After 72 h, the vascularization of CAM was evaluated. The vessel density was defined as the proportion of vessel area over the total area measured. The photographs of CAM blood vessels formation inside of rings were obtained using a stereomicroscope Olympus SZ61 (Tokyo, Japan) and digital camera (PROMICRA 3.2, Prague, Czech Republic). Subsequently, photographs were processed using QuickPHOTO MICRO microscope software version 3.2 (Promicra, Prague, Czech Republic). The experiments were repeated three times with 10 eggs per condition. Representative images were analyzed using Wimasis WimCAM web-based service (Wimasis GmbH, Munich, Germany) to quantify angiogenesis.

### 2.9. Alcian Blue Staining of CAM Membrane

Paraffin CAM sample sections of 7 μm thickness were deparaffinized and dehydrated to distilled water followed by staining in alcian blue solution (Sigma-Aldrich St. Louis, MO, USA), 1% in 3% acetic acid, pH 2.5) for 60 min at 50 °C. Sections were rinsed in running tap water and then counterstained with Mayer’s hemalum solution (Merck Millipore, Burlington, MA, USA) for 5 min. Subsequently, sections were rinsed in running tap water and stained with eosin for 30 s. Samples were rehydrated, cleared, and mounted in Entellan (Merck Millipore, USA).

### 2.10. Statistical Analyses

All values were expressed as mean ± SD (standard deviation). Statistical analyses of the data were performed using standard procedures, with one-way analysis of variance (ANOVA) followed by the Student *t*-test. Differences were considered significant when *p* < 0.05. Throughout this paper * indicates *p* < 0.05, ** *p* < 0.01, *** *p* < 0.001 versus untreated control; # *p* < 0.05, ## *p* < 0.01, ### *p* < 0.001 versus VEGF alone; $ *p* < 0.05, $$ *p* < 0.01, $$$ *p* < 0.001 versus bFGF alone.

## 3. Results

### 3.1. Analysis of Usnic Acid

**Figure 1:**
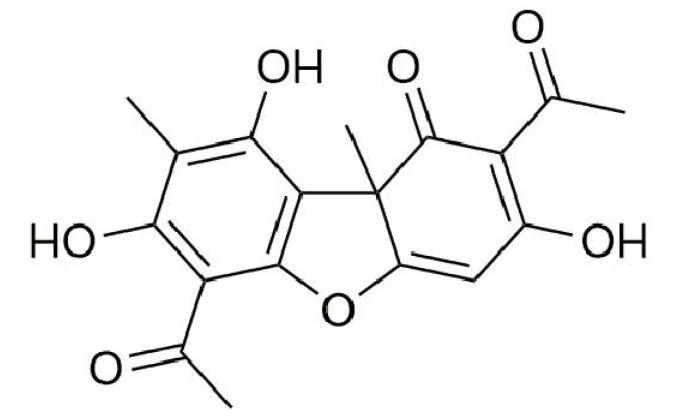
Chemical structure of usnic acid.

The isolated fraction from *Usnea antarctica* was identified by TLC and HPLC as dibenzofuran usnic acid (Figure 1). The amount of 50 mg of UA was dissolved in 1 ml DMSO (100%) and stored at −20 °C. The final concentrations (10, 50, 100 μM) of UA were prepared just before the use. The final concentration of DMSO was <0.2%.

The structure of usnic acid was established by 1D and 2D NMR spectra (Figure 1, Figures S1 and S2). The application of 2D heteronuclear ^1^H–^13^C (HSQC, HMBC) experiments made it possible to unambiguously assign all signals in the ^1^H and ^13^C NMR spectra. Moreover, ^1^H and ^13^C NMR spectra were compared with NMR spectra of usnic acid commercial standard. The ^1^H and ^13^C NMR chemical shifts in DMSO-d_6_ for isolated usnic acid are given in Table S1. The purity of isolated UA was 96%.

### 3.2. VEGF and bFGF Promote HUVEC Proliferation

**Figure 2:**
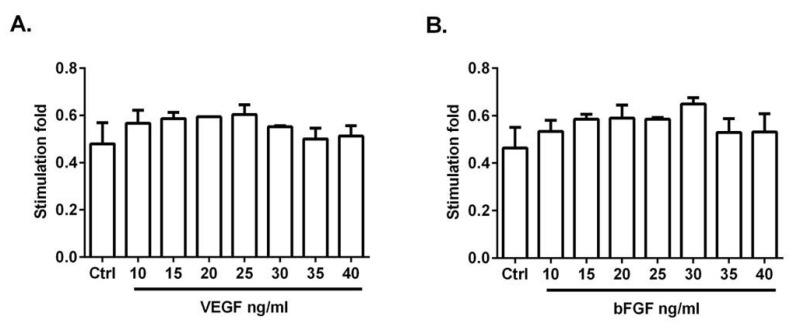
VEGF and bFGF promote HUVECs proliferation. Cells were treated with different concentrations (10–40 ng/mL) of VEGF (**A**) or bFGF (**B**) for 48 h, and cell viability was detected by MTT assay. Each treatment was performed in triplicate and repeated three times.

To determine the optimal dose of both factors to promote endothelial cell proliferation, HUVECs were treated with 10–40 ng/mL of VEGF or bFGF for 48 h before the assays. HUVEC proliferation was increased most at 25 ng/mL of VEGF (Figure 2A) and 30 ng/mL of bFGF (Figure 2B). The stimulation fold was not further increased by a higher concentration of both factors. In the following experiments, 25 ng/mL of VEGF and 30 ng/mL of bFGF were used.

### 3.3. MTT Assay

**Figure 3:**
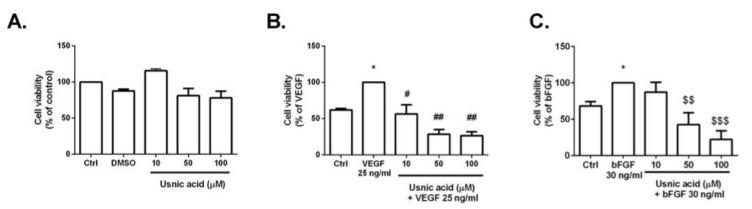
Inhibitory effect of usnic acid (UA) on cell viability. HUVECs cells were treated with vehicle (DMSO) and various concentrations of UA (10, 50, 100 μM) in the absence (**A**) or presence (**B**) of VEGF (25 ng/mL) and (**C**) bFGF (30 ng/mL) for 48 h. Values are means ± SD from three independent experiments (* *p* < 0.05 versus control; # *p* < 0.05; ## *p* < 0.01 versus VEGF alone; $$ *p* < 0.01; $$$ *p* < 0.001 versus bFGF alone).

Angiogenesis is a complex biological process that requires the precise coordination of proliferation, migration, and tube formation by endothelial cells, where vascular endothelial growth factor (VEGF) and basic fibroblast growth factor (bFGF) play important roles. The cytotoxic activity of UA was studied by the colorimetric MTT assay as described in Materials and Methods. UA was dissolved in DMSO. The final concentration of DMSO in the culture medium was <0.2% and exhibited no cytotoxicity, as seen in Figure 3A. As displayed in Figure 3B, UA significantly reduced the cellular viability of VEGF-stimulated HUVECs after 48 h with an IC_50_ value of 33.57 ± 1.33 μM. Similarly, UA was able to significantly inhibit bFGF-stimulated HUVECs after 48 h with IC_50_ 44.58 ± 4.2 μM (Figure 3C). There was no significant toxic effect of UA on HUVECs alone (IC_50_ > 100 μM), as displayed in Figure 3A. For the following experiments, the UA concentration taken from VEGF/UA analyses (33.57 μM) was used.

### 3.4. Usnic Acid Inhibited the Migration of VEGF- and bFGF-Stimulated HUVECs

**Figure 4:**
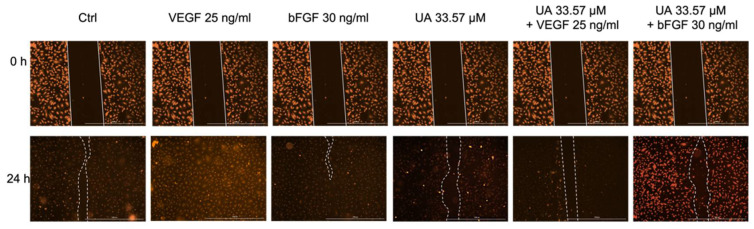
Influence of IC_50_ of usnic acid (UA) alone or co-incubated with VEGF (25 ng/mL) or bFGF (30 ng/mL) on the migration of HUVECs. Representative images of HUVECs scratch wound assay are shown for the 0 h and 24 h time points (100× magnification, scale bar 1000 μm).

The anti-migratory activity of UA on HUVECs was determined by the scratch wound-healing assay. As seen in Figure 4, the HUVECs migrated into the wounded area after 24 h incubation with VEGF and bFGF. VEGF and bFGF-stimulated HUVECs migration was partially inhibited by UA (33.57 μM) treatment after 24 h. UA alone also inhibited the migration of endothelial cells compared to the control.

### 3.5. Usnic Acid Blocked the Formation of HUVECs Tubule in the Presence of VEGF and bFGF

**Figure 5:**
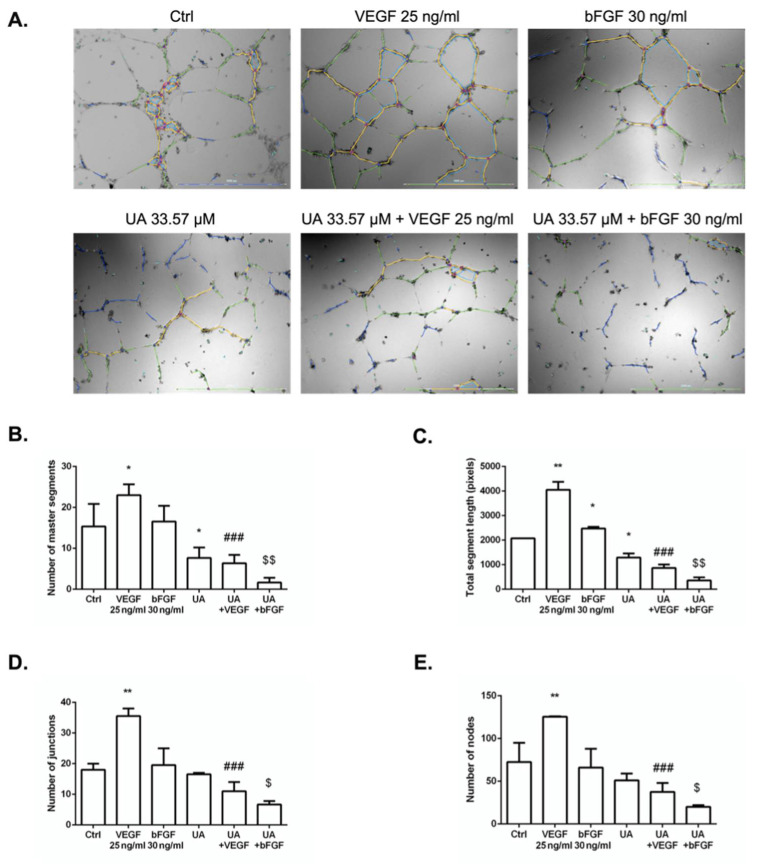
Effects of usnic acid (UA, 33.57 μM) on tube formation. (**A**) Representative micrographs of the human umbilical vein endothelial cells (HUVECs) tube formation showing network formation after 24 h treatment (100× magnification, scale bar 1000 μm). Graphs show automatic parameter detection by ImageJ software. Tube segments are colored in yellow, green, and dark blue; the master junction in pink; and nodes surrounded by junction in red surrounded by blue. Quantification of the number of master segments (**B**), total segment length (**C**), number of master junctions (**D**), and number of nodes (**E**). Data are expressed as mean ± SD of three independent experiments (* *p* < 0.05; ** *p* < 0.01 versus control; ### *p* < 0.001 versus VEGF alone; $ *p* < 0.05; $$ *p* < 0.01 versus bFGF alone).

The anti-angiogenic activity of UA was investigated using an endothelial tube formation assay. Tube-like structures harboring branches, segments, junctions, and nodes were observed after culturing HUVECs on Matrigel for 24 h (Figure 5A). The pro-angiogenic factors VEGF and bFGF increased tube formation versus vehicle, as expected. UA was able to significantly inhibit all tested parameters in VEGF- and bFGF-stimulated HUVECs (Figure 5B–E), while UA in the absence of VEGF or bFGF inhibited only the number of master segments and total segment length compared to non-stimulated HUVECs (Figure 5B,C).

### 3.6. Fibrin Bead Sprouting Assay

**Figure 6:**
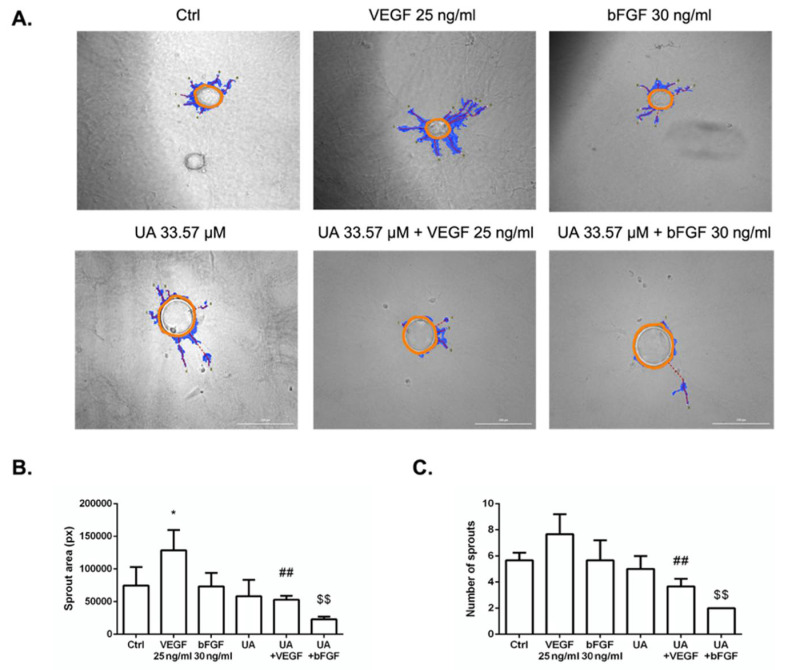
Fibrin gel bead assay to evaluate the anti-angiogenic potential of usnic acid (UA). (**A**) Representative images of HUVEC-coated beads embedded in a fibrin gel in the absence or presence of VEGF (25 ng/mL) or bFGF (30 ng/mL) treated with IC_50_ of UA photographed at day 7 (100× magnification; scale bar 200 μm). Graphs representing sprout area (px) (**B**) and number of sprouts (**C**). In all graphs, values are given as average ± SEM (*n* = 3 per condition). * *p* < 0.05 compared to control; ## *p* < 0.01 compared to VEGF alone; $$ *p* < 0.01 compared to bFGF alone.

A 3D angiogenic sprouting assay was performed to analyze the effect of UA on VEGF- and bFGF-induced angiogenic sprouting. When HUVECs were placed into a fibrin gel assay and cultured with VEGF or bFGF, only VEGF significantly promoted endothelial cell vessel formation, as seen in Figure 6A. The sprouting ability of HUVECs in the presence of UA co-incubated with growth factors was blocked on day 7. This deficiency was confirmed by reduced sprouting area and number of sprouts using the WimTube webtool (Figure 6B,C).

### 3.7. The Anti-Angiogenic Effect of UA on Vascularization of Quail CAM Model

**Figure 7:**
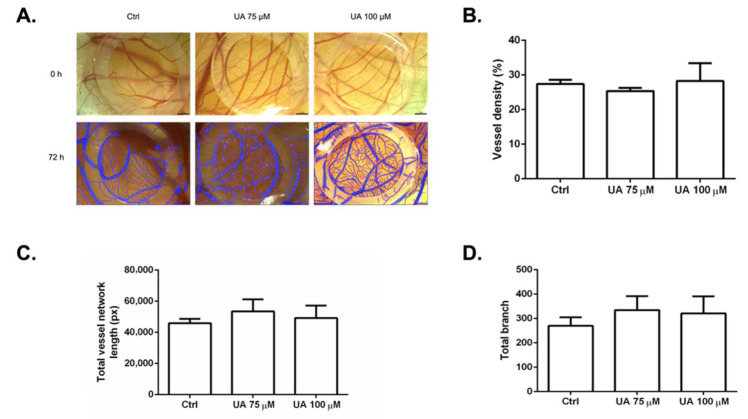
The effects of usnic acid (UA) alone on capillary formation ex ovo in the quail vascular system. (**A**) Representative images of CAMs at 0 h and 72 h after incubation with UA (75 μg/mL, 100 μg/mL). The results are summarized in the graphs as vessel density (**B**), total vessel network length (**C**), and total branch (**D**). Each group contained 10 CAMs, and the experiment was repeated three times.

To determine whether our in vitro findings would be applicable ex ovo, we performed the CAM assay, which is the most frequently used model to screen anti-angiogenic properties more efficiently. The anti-angiogenic effect of the tested compound was evaluated through image analysis using the WimCam software program (Figure 7A), where inhibition of angiogenesis was evaluated based on three parameters: percentage of vessel density, total vessel network length, and sprouting ability indicated by total branching.

In the preliminary experiments, UA in a concentration of 33.57 μM had no significant effect on CAM vascularization (data not shown). Therefore, we used concentrations of 75 μM and 100 μM, which showed significant differences. UA (75 μM, 100 μM) in the absence of VEGF or bFGF did not reduce the formation of blood vessels, as seen in Figure 7A–D. UA displayed no damage to blood vessels or embryo viability. Symptoms such as hemorrhage, hyperemia, or coagulation were also not observed, which points to no signs of toxicity of UA alone.

**Figure 8:**
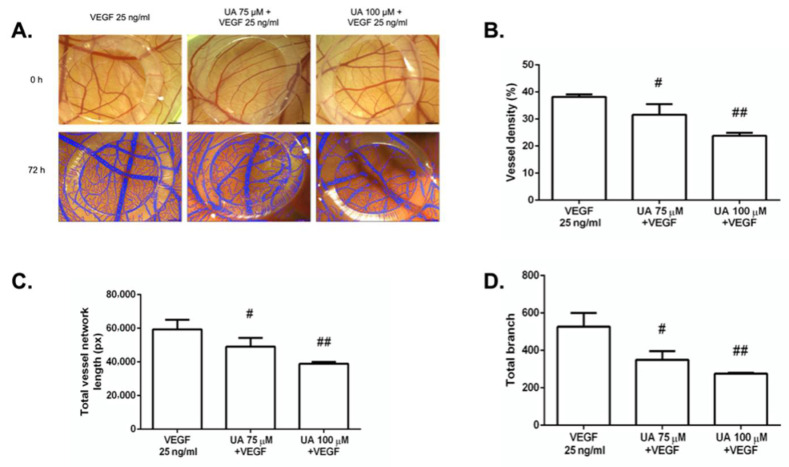
Usnic acid (UA) reduced capillary formation ex ovo in the VEGF-stimulated quail vascular system. (**A**) Representative images of CAMs at 0 h and 72 h after incubation with UA (75 μM, 100 μM) in combination with VEGF (25 ng/mL). The results are summarized in the graphs as vessel density (**B**), total vessel network length (**C**), and total branch (**D**). Each group contained 10 CAMs, and the experiment was repeated three times. Error bars represent ± SD (# *p* < 0.05; ## *p* < 0.01 versus VEGF alone).

To monitor the effect of UA on VEGF-induced angiogenesis, the vascular formation was supported by the vascular stimulator VEGF. Endogenously supplied VEGF caused marked increased vessel density, total vessel network length, and total branching (Figure S1). UA at concentrations of 75 μM and 100 μM was able to significantly block the VEGF-induced vessel growth in all three parameters (Figure 8B–D). The results also indicate the dose-dependence of the tested substance.

**Figure 9:**
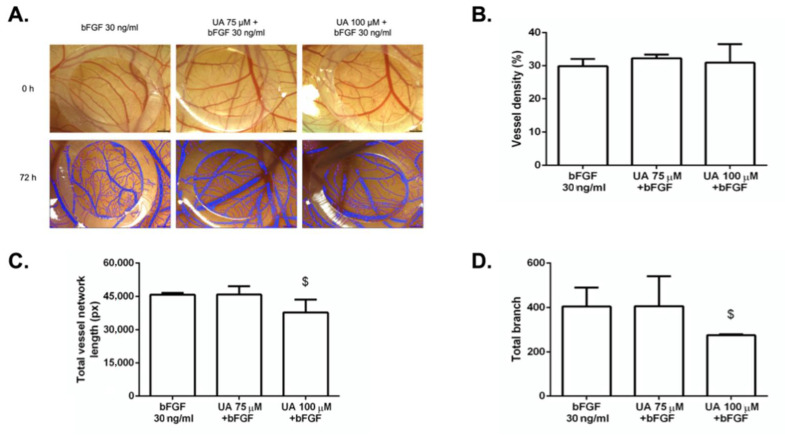
Usnic acid (UA) reduced capillary formation ex ovo in the bFGF-stimulated quail vascular system. (**A**) After incubation for 72 h, CAMs were photographed with a digital camera. Each group contained 10 CAMs, and the experiment was repeated three times. The results are summarized in the graphs as vessel density (**B**), total vessel network length (**C**), and total branch (**D**). Error bars represent ± SD ($ < 0.05 versus bFGF alone).

In contrast to VEGF, bFGF affected CAM vascularization more slightly (Figure S3). As shown in Figure 9, UA at the concentration of 100 μM significantly reduced total vessel network length and branch point formation (Figure 9C,D) in the quail CAM when compared with bFGF, whereas we did not observe any difference using 75 μM of UA. Furthermore, vessel density was also unaffected when UA 100 μM was used.

### 3.8. Histological Examination of CAM Treated with Usnic Acid

**Figure 10:**
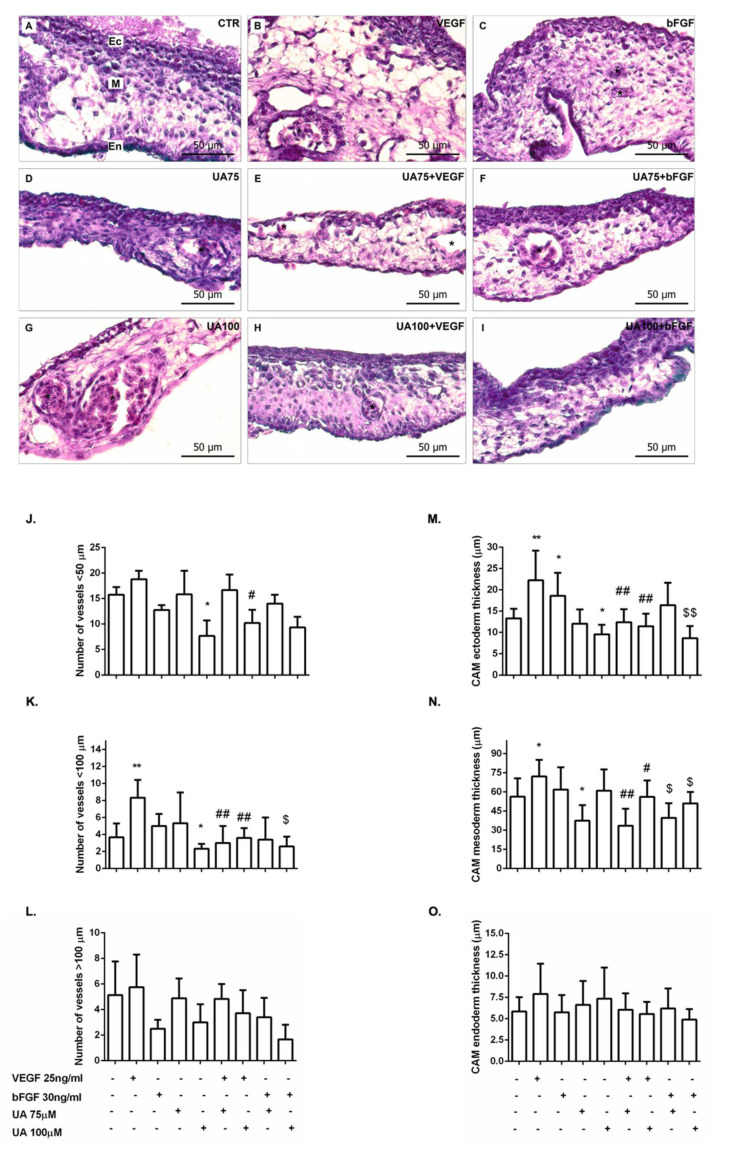
H–E/alcian blue staining of chorioallantoic membranes showing the chorionic epithelium (ectoderm, Ec), the intermediate vascularized mesenchyme (mesoderm, M) with vessels (asterisks), and the deep allantoic epithelium (endoderm, En). Evaluation of CAM tissue response to 0.9% NaCl (**A**), VEGF 25 ng/mL (**B**), bFGF 30 ng/mL (**C**), UA 75 μM (**D**), UA 75 μM + VEGF (**E**), UA 75 μM + bFGF (**F**), UA 100 μM (**G**), UA 100 μM + VEGF (**H**), and UA 100 μM + bFGF (**I**) on ED9. Original magnification 40×, scale bar 50 μm. Graphs represent number of vessels <50 μm (**J**), <100 μm (**K**), >100 μm (**L**) and thickness of ectoderm (**M**), mesoderm (**N**), and endoderm (**O**). Error bars (* *p* < 0.05, ** *p* < 0.01 versus control; # *p* < 0.05, ## *p* < 0.01 versus VEGF alone; $ *p* < 0.05, $$ *p* < 0.01 versus bFGF alone).

The inhibitory activity of UA was confirmed by histological observations. Saline-treated control embryos showed three layers of CAM, i.e., ectoderm, mesoderm, and endoderm (Figure 10A). We observed an increase in vascular area with an increase in numerous blood vessels < 100 μm in diameter after application of VEGF alone (Figure 10B,K). Thickness at the ectoderm and mesoderm layers increased as well (Figure 10M–N). However, vascularization of embryos co-incubated with bFGF alone did not show any significant change in the number of blood vessels (Figure 10C,J–L). CAM ectoderm thickness was marginally affected (Figure 10M).

Treatment with UA alone (Figure 10D,G) at 72 h of CAM development induced an inhibitory response in blood vessels <50 and <100 μm in diameter when only using the highest concentration (100 μM) (Figure 10J,K). Moreover, CAM ectoderm and mesoderm reduction followed (Figure 10M–N).

CAM treated with various concentrations of UA in the presence of VEGF (Figure 10E,H) showed inhibition of small and major vessels (Figure 10J,K), with a significant decrease in tissue thickness of ectoderm and mesoderm when compared to the control (Figure 10M,N).

Although bFGF did not alter the number of blood vessels, co-incubation with UA (100 μM) (Figure 10I) showed a reduction in the number of blood vessels < 100 μm in diameter (Figure 10K) as well as a reduction in ectodermal and mesodermal tissue thickness (Figure 10M,N). UA (75 μM) co-incubated with bFGF (Figure 10F) caused no significant difference in the number of vessels (Figure 10J–L). The thickness of the mesoderm was marginally reduced (Figure 10N).

The outermost layer, the endoderm, was not affected by UA alone or in the presence of VEGF or bFGF (Figure 10O).

## 4. Discussion

Angiogenesis is a sophisticated multistep process that involves the migration, proliferation, and differentiation of endothelial cells regulated by numerous growth factors and many intracellular pathways. Uncontrolled growth of blood vessels contributes to the progression of malignant tumors and the formation of metastasis. Therefore, inhibition of angiogenesis is an alternative therapeutic strategy for the treatment of a wide range of cancers [35,36,37,38].

However, current anti-angiogenic drugs have been shown to have limited clinical benefits. The most serious limitation of anti-angiogenic treatment is the development of resistance to anti-angiogenic drugs [39,40], which can happen through a variety of mechanisms, ranging from early-stage resistance to late-stage adaptation to the microenvironment [41,42]. Although vascular endothelial growth factor is considered the best known angiostimulatory factor, many other growth factors can trigger the process of angiogenesis [43,44]. These factors, including fibroblast growth factors (FGFs), epidermal growth factors (EGFs), hepatocyte growth factors (HGFs), and placental growth factors (PIGFs), may be responsible for the development of secondary resistance [43,44,45].

Among the many potential alternatives for treating cancer, much more attention is currently being paid to anti-cancer agents from natural sources. Tremendous progress by the scientific community has been made bringing natural products into clinical use, which has opened new therapeutic opportunities for the treatment of cancer [46,47,48].

Secondary metabolites of lichens have been considered as unique natural molecules because of their distinctive biological activities [18]. Therefore, the present study was designed to analyze the potential anti-angiogenic effect of usnic acid (UA) on VEGF- and bFGF-induced angiogenesis.

The antiproliferative activity of UA was firstly reported on Lewis lung carcinoma cells in the 1970s [28]. Later, it was published that the (−) and (+) isomers of UA showed moderate to strong cytotoxicity against a wide range of mouse and human tumor cell lines in vitro (A2780, MCF-7, SKBR-3, HT-29, HCT116, HL-60, Jurkat, HeLa, DU-145, PC-3, and HTB-140) in the IC_50_ range of 48.5–199.2 µM [27,49,50].

According to Koparal et al. [21], isolated UA reduced the viability of HUVEC cells in a dose-dependent manner with IC_50_ values of 217.31 ± 3.51 (24 h) and 52.18 ± 1.71 (48 h). Other studies indicated that UA significantly reduced HUVEC cell viability when the IC_50_ was less than 10 µM [51] or when the IC_50_ value was below 20 µM [52]. All of the authors utilized commercially available UA.

In comparison, our results obtained from the MTT screening assay indicated that UA isolated from *Usnea antarctica* in combination with VEGF (25 ng/mL) had a cytotoxic effect on HUVEC cells with an IC_50_ of 33.57 ± 1.33 µM, whereas the results obtained from non-stimulated HUVECs indicated no cytotoxic effect at the maximum tested concentration of 100 μM. Furthermore, this is the first study that indicates the ability of UA to also reduce bFGF-stimulated HUVEC cell viability, with an IC_50_ 44.58 ± 4.2.

In addition to the proliferation of endothelial cells, the migration and tube formation of endothelial cells are other important steps of angiogenesis [38]. The wound-healing mechanisms of UA, as well as its inhibitory activity on tube formation, were described by a few authors. Song et al. [17] tested to the effect of commercial UA on these processes of angiogenesis. The authors demonstrated its inhibitory effect on migration and the formation of tubular structures of HUVECs in a dose-dependent manner (1, 10, 20 µM). Inhibition of several signaling pathways involved in the stimulation of angiogenesis by UA was also reported, as well as the inhibition of VEGF-induced corneal blood vessel formation in mice. Koparal [21] investigated the inhibitory effect of UA isolated from *Cladonia foliacea* on endothelial cell proliferation and vascular formation. His study showed a potential inhibitory effect of the tested compound on these processes in a dose-dependent manner using a minimum concentration of 71.5 µM. Other secondary metabolites of lichens, such as barbatolic acid, vulpinic acid, olivetoric acid, emodin, secalonic acid D, and parietin, were identified to have an anti-angiogenic effects. The authors demonstrated their inhibitory effects on the basic steps of angiogenesis processes, such as cell proliferation, tube formation, and cell migration, in the range of concentrations of 5–100 μM [16,19,20,53].

Our observations demonstrated that the effects of UA from *U. antarctica* on HUVEC cells are consistent with results published by Song [17] and Koparal [21]. Incubation of endothelial cells with 33.57 µM of UA led to the inhibition of VEGF- and bFGF-mediated migration in vitro. Furthermore, the application of UA decreased the number of master segments, total segment length, number of master junctions, and number of nodes in both experimental conditions, namely, UA co-incubated with VEGF and UA co-incubated with bFGF. However, the combination of UA with VEGF had a higher inhibitory efficiency. Furthermore, we also observed that UA decelerates sprouting of HUVECs during early phase angiogenesis in the fibrin gel bead assay and thus confirmed its anti-angiogenic potential.

There is no doubt that using ex ovo or in vivo experiments provide us with a fundamental bridge of the gap between in vitro findings and clinical applications. Among various in vivo model systems designed to study the process of angiogenesis, avian embryo models are useful tools for analyzing the angiogenic and anti-angiogenic potential of multiple substances [54]. The avian chorioallantoic membrane (CAM) model includes all steps of angiogenesis and vascular maturation to form functional vascular networks [55]. The quail CAM model was used in our study to confirm the anti-angiogenic effects of UA and to obtain more physiologically relevant results, which permits us to develop a more accurate experimental conclusion.

CAM is a specialized, highly vascularized avian embryo tissue that serves as an ideal indicator of the anti- or pro-angiogenic properties of tested compounds [54,56]. Structurally, CAM is made up of three layers, which include the ectoderm, the mesoderm, and the endoderm [56]. A rich vascular system develops within the mesoderm that is the largest component of CAM [57,58]. In 2012, Song et al. [17] performed a study using an in vivo VEGF-induced CAM model to study the anti-angiogenic potential of commercial (+)-UA The authors reported that UA (1 μg/disc) significantly blocks the formation of new blood vessels in the CAM assay. Similarly, Kwak et al. [59] examined the effects of secondary metabolite emodin on angiogenesis in vivo using the CAM assay. A combination of emodin (50 μM) and VEGF-A suppressed vessel formation.

In comparison with our in vitro results, we observed the anti-angiogenic activity of UA on CAM’s vascularization at a 2-fold higher concentration (33.75 μM vs. 75 μM). There were no significant differences in stimulation or inhibition of VEGF- or bFGF-induced vascularization using IC_50_ UA calculated based on the in vitro MTT assay. There can be several explanations for using a higher concentration in the ex ovo model. The 3D models are much better biomimetic angiogenesis tissue models that resemble more closely the in vivo conditions [55]. In adherent 2D cultures, cells must adapt to a hard and flat surface, most commonly in culture flasks with a medium as a source of nutrition. Their access to nutrients as well as to the therapeutic agents is significantly different from that to which they would be exposed in living tissue or a 3D model [60,61]. It should be noted that any topically applied component must pass through the outer epithelium and reach the vessels located in the mesodermal layer.

Our results indicated the ability of UA isolated from *Usnea antarctica* to suppress CAM angiogenesis in a dose-dependent manner using concentrations of 75 μM and 100 μM. Statistical analysis of three parameters, including vessel density, total vessel length, and vascular branching, showed the strongest potency of VEGF as an angiogenesis-promoting factor compared to bFGF. Subsequently, the UA significantly inhibited the VEGF-induced angiogenesis in a dose-dependent manner. The inhibitory effect of UA on bFGF-induced angiogenesis was lower, suggesting the strongest affinity of UA to the vascular endothelial growth factor.

To confirm the results obtained from the previous methodology, a histological section of individual chorioallantoic membranes was performed. Histologically, CAM comprises three membrane layers, as mentioned above. Blood vessels <50 μm, <100 μm, and >100 μm in diameter and the thicknesses of all layers of CAM were closely examined. To the best of our knowledge, there is no study that has performed this method using a secondary metabolite of lichens as a potential anti-angiogenic agent.

According to the results obtained from hematoxylin–eosin and alcian blue staining of histological sections of CAM, UA decreased the formation of blood vessels in the mesoderm, especially in vessels with diameters <100 μm. This effect was also demonstrated by the reduction in the thickness of the mesoderm in which the formation of blood vessels takes place. Similarly, the thickness of the ectoderm was reduced. Galanty et al. [62] demonstrated that UA has a strong penetrating potential, which may result from its lipophilic properties [63]. However, our observations revealed no change in endoderm thickness. The reason may be insufficient penetration of the UA into the outermost layer or a short biological time. This hypothesis requires more investigation.

The structures of the tested natural compounds and their purities determine bioavailability, effectiveness, and efficiency. UA showed several promising effects, as mentioned previously. However, it must be noted that such molecules can be affected by the phenomenon PAIN (Pan-Assay Interference Compounds), which came to attention in 2010 [64]. Due to the nature of these molecules, they can interfere with screenings results by metal chelating, membrane disturbing, and by non-specific protein interaction. Several molecules from the PAIN list belong to polyphenol groups [65]. From the point of view of UA (CAS No.: 7562-61-0), it belongs to the dibenzofuran derivatives, and with three OH groups, it is also classified into the group of polyphenols. Even though UA has not yet been included in PAINS (data not available) and we have not tested it either, it is necessary to pay attention to this possibility, and it will be necessary to check it in the future.

In conclusion, due to the weaker efficacy and the emergence of resistance to current anti-VEGF drugs, it is important to focus research on substances capable of inhibiting more than one molecule of angiogenesis, or a combination of these factors that could act synergistically. The research about this process continues, and it will be necessary to carry out further experiments in the future, which will provide us with more detailed information about the possible mechanism of action of UA in the field of angiogenesis as well as of cancer.

## 5. Conclusions

Taken together, the present study shows that the secondary metabolite usnic acid isolated from *Usnea antarctica* is a promising anti-angiogenic agent that inhibits VEGF- and bFGF-induced angiogenesis both in vitro and ex ovo. Anti-angiogenic activity of usnic acid is mediated via the inhibition of migration, proliferation, and tube formation of endothelial cells as well as by blocking the formation of new blood vessels in quail embryo. The CAM assay, as an alternative animal model and a gap between in vitro and in vivo models, provided us with more accurate experimental conclusions. However, further studies using animal models are needed to support the clinical use of usnic acid in chemoprevention or cancer treatment.

## Data Availability

The data presented in this study are available in the Supplementary Materials or can be provided by the authors.

## Supplementary Materials

The following supporting information can be downloaded at: https://www.mdpi.com/article/10.3390/life12091444/s1, Figure S1: 1H (600 MHz, DMSO-d6) and 13C (150 MHz, DMSO-d6) NMR spectra of usnic acid; Figure S2: HSQC and HMBC spectra of usnic acid; Figure S3: Proangiogenic effect of VEGF (25 ng/mL) and bFGF (30 ng/mL) on CAM vascularization; Table S1: 1H and 13C NMR chemical shifts of isolated usnic acid in DMSO-d6.
