# Supplementary material for: Usnic Acid Isolated from Usnea antarctica (Du Rietz) Reduced In Vitro Angiogenesis in VEGF- and bFGF-Stimulated HUVECs and Ex Ovo in Quail Chorioallantoic Membrane (CAM) Assay
Source: Life (Basel). 2022 Sep 16;12(9):1444. doi: 10.3390/life12091444 (PMC9503005; doi:10.3390/life12091444)
Supplement: Supplementary file 1 [file life-12-01444-s001.zip › life-1883105-supplementary.pdf]

Supplementary Materials

Usnic Acid Isolated from *Usnea antarctica* (Du Rietz) Reduced In Vitro Angiogenesis in VEGF- and b-FGF-Stimulated HUVECs and Ex Ovo in Quail Chorioallantoic Membrane (CAM) Assay

Klaudia Petrová[1*], Miriam Bačkorová[2], Zuzana Demčišáková[3], Eva Petrovová[3], Richard Frenák[4], Michal Goga[4], Martin Bačkor[4,5], Ján Mojžiš[1], and Martin Kello[1*]

[1] Department of Pharmacology, Faculty of Medicine, Pavol Jozef Šafárik University, 040 01 Košice, Slovakia
[2] Department of Pharmaceutical Technology, Pharmacognosy, and Botany, University of Veterinary Medicine and Pharmacy, 041 81, Košice, Slovakia
[3] Department of Anatomy, Histology, and Physiology, University of Veterinary Medicine and Pharmacy, 041 81 Košice, Slovakia
[4] Department of Botany, Institute of Biology and Ecology, Faculty of Science, Pavol Jozef Šafárik University, Mánesova 23, 041 67, Košice, Slovakia
[5] Institute of Biotechnology, Faculty of Biotechnology and Food Sciences, Slovak University of Agriculture in Nitra, Trieda Andreja Hlinku 2, 949 76, Nitra, Slovakia; [*] Correspondence: claudy.petrova@gmail.com (K.P.); kellomartin@yahoo.com (M.K.)

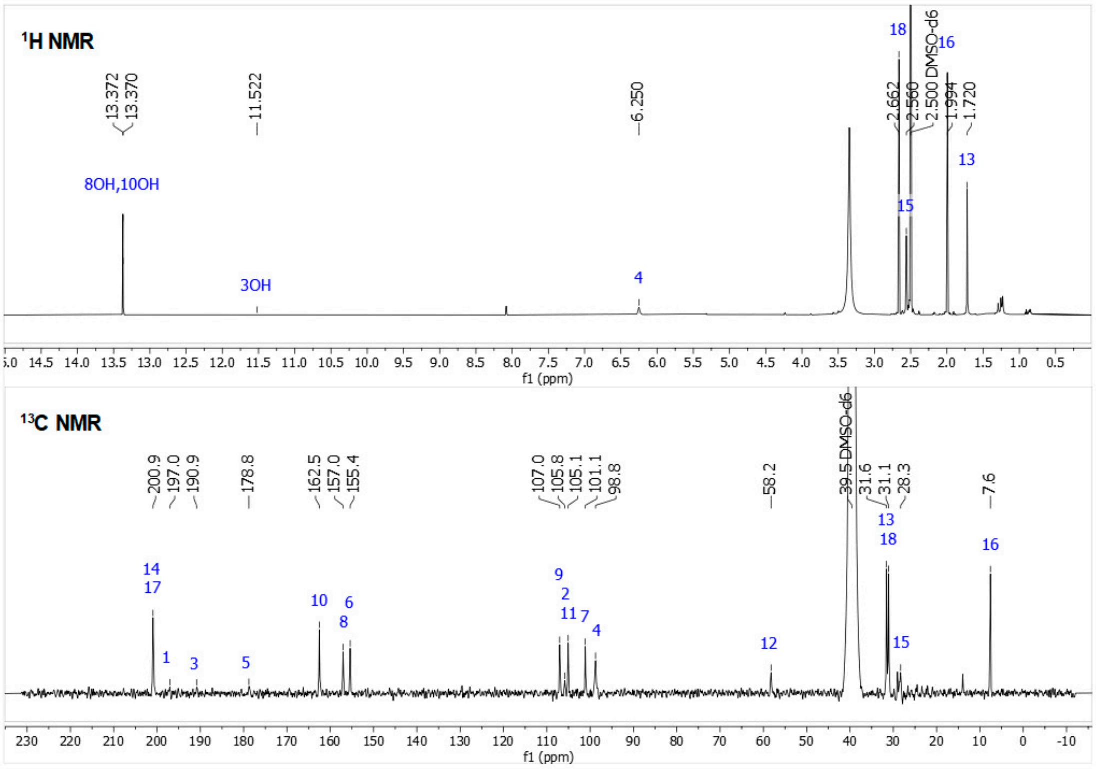

Figure S1. [1]H (600 MHz, DMSO-d6) and ^{13}C (150 MHz, DMSO-d6) NMR spectra of usnic acid.

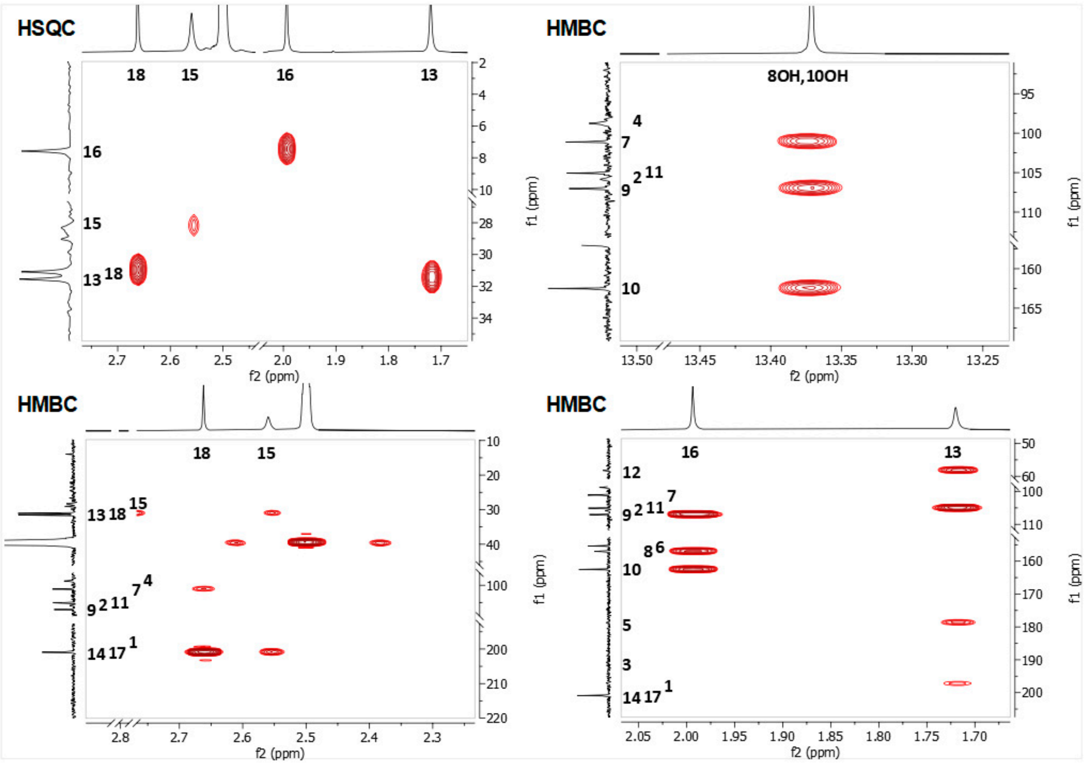

Figure S2. HSQC and HMBC spectra of usnic acid.

Table S1. ^{1}H and ^{13}C NMR chemical shifts of isolated usnic acid in DMSO-d6.

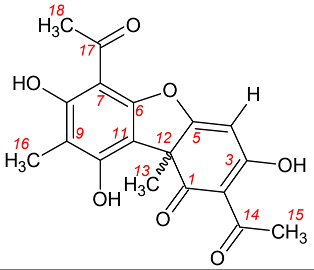

No.	δ_H [ppm]	δ_C [ppm]	HMBC correlations
1		197.0	
2		105.8	
3	11.33*	190.9	
4	6.31	98.8	
5		178.8	
6		155.4	
7		101.1	
8	13.37*	157.0	101.1 (C-7), 107.0 (C-9)
9		107.0	
10	13.37*	162.5	
11		105.1	
12		58.2	
13	1.74	31.6	58.2 (C-12), 105.1 (C-11), 178.8 (C-5), 197.0 (C-1)
14		201.0	
15	2.59	28.3	105.8 (C-2), 201.0 (C-14)
16	2.00	7.6	107.0 (C-9), 157.0 (C-8), 162.5 (C-10)
17		201.0	
18	2.67	31.1	101.1 (C-7), 201.0 (C-17)

* ^{1}H chemical shift of OH proton

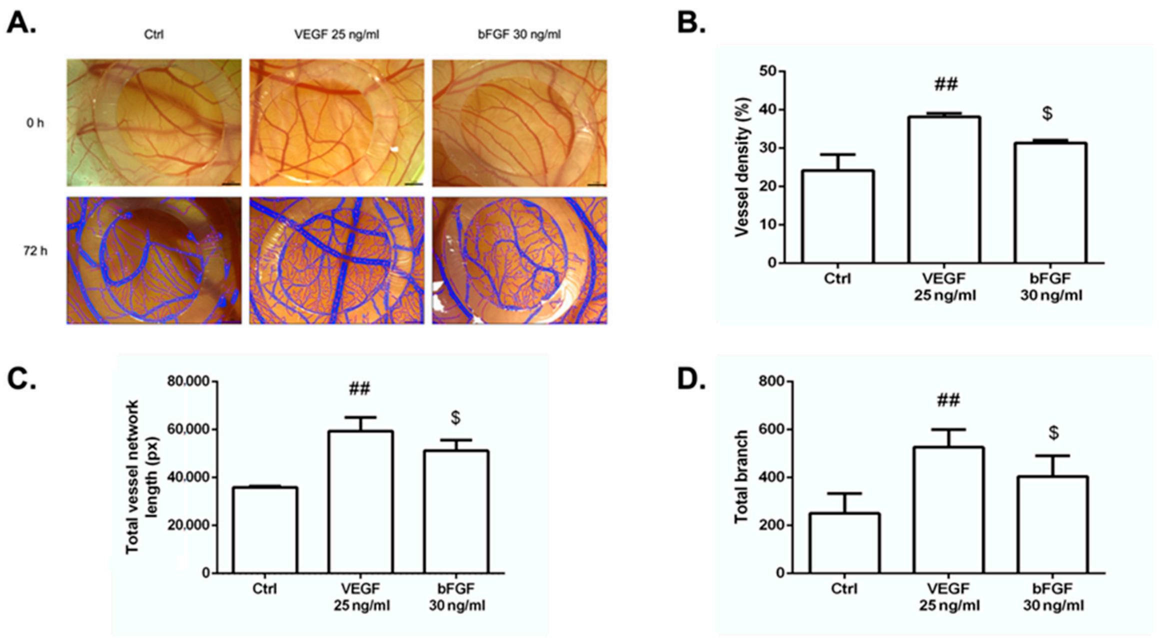

Figure S3: Proangiogenic effect of VEGF (25ng/ml) and bFGF (30ng/ml) on CAM vascularization (**A**). The results are summarized in the graphs as vessel density (**B**), total vessel network length (**C**), and total branch (**D**). Each group contained 10 CAMs and the experiment was repeated three times. Error bars represent ± SD (##$p < 0.01$ versus VEGF; \$$p < 0.05$ versus bFGF).
